# P36 Letting the cat out of the bag: delafloxacin as an oral option for the treatment of a complex wound infection

**DOI:** 10.1093/jacamr/dlaf118.043

**Published:** 2025-07-14

**Authors:** John Cunniffe

**Affiliations:** Countess of Chester Hospital NHS Foundation Trust, Chester, UK

## Abstract

**Background:**

Complex wound infections can be challenging to treat and often necessitate IV antimicrobial therapy, typically delivered in inpatient settings or via outpatient parenteral antimicrobial therapy (OPAT) services. With growing pressures across healthcare systems, strategies facilitating early discharge through effective and simple oral treatment regimens are preferred. Delafloxacin, a fluoroquinolone licensed for the treatment of acute bacterial skin and skin structure infections (ABSSSI) and community-acquired pneumonia (CAP) in adults, offers a convenient oral dosing regimen and has broad-spectrum coverage, good tissue and biofilm penetration, and enhanced efficacy in acidic environments characteristic of infected wounds. Together, these properties make delafloxacin a promising option for the treatment of complex wound infections. We describe a case of off-label use of delafloxacin to resolve a complex polymicrobial wound infection yielding *Pasteurella multocida* and anaerobes, which resulted from a cat bite, and had been unresponsive to prior therapy.

**Case description:**

A 55-year-old female was admitted to the hospital with a soft tissue infection of the lower right leg, as a result of a recent cat bite. The patient's wound required surgical debridement. Samples taken on admission and in theatre isolated *P. multocida* plus anaerobes. She was discharged home on oral co-amoxiclav. Following ongoing purulent discharge and inflammation, she was re-admitted. Her therapy was converted to IV ceftriaxone and metronidazole. She was slow to improve and was referred for debridement. Samples continued to isolate the same organisms. She was discharged home on oral doxycycline and metronidazole. Due to the lack of clinical response before finishing the further course of treatment, off-label use of delafloxacin was discussed, owing to its favourable pharmacological properties, including activity against the repeatedly isolated pathogens. The patient was converted to oral delafloxacin 450mg twice daily. Within 3 days, symptoms had improved, with reduced inflammation; after 7 days, further improvement was noted, blood parameters returned to normal levels, although there was still some purulent discharge. However, cultures showed no growth. Following a further week of delafloxacin, the patient continued to improve, with no pus evident. At the next review, inflammation had fully resolved, the patient was weight-bearing, and well enough to resume normal activities.

**Conclusions:**

In this case, oral delafloxacin led to the rapid clinical resolution of a complex wound infection yielding *P. multocida* and anaerobes, resulting from a cat bite, and showed a poor prior response to licensed agents, despite sensitivity testing indicating susceptibility. The successful outcome of delafloxacin treatment could have been contributed to by its pharmacological properties and ease of administration, facilitating patient compliance. Furthermore, delafloxacin’s potential to facilitate early hospital discharge on a simple oral regimen could help to ease the pressures experienced by hospitals and OPAT services, whilst supporting antimicrobial stewardship objectives. Nevertheless, further evaluation is needed to help define its possible role in managing complex wound infections beyond its current license; it is essential that use is guided by appropriate governance frameworks.

**Transparency declarations:**

Medical writing support was funded by Menarini group, manufacturer of delafloxacin.

